# Rhino-orbito-cerebral mucormycosis after allogeneic hematopoietic stem cell transplantation for very severe aplastic anemia in a child: a case report

**DOI:** 10.3389/fped.2025.1529656

**Published:** 2025-04-14

**Authors:** Yimei Ma, Ziting Xia, Bochao Cheng, Bo Wang, Xingming Huang, Xiaoxi Lu

**Affiliations:** ^1^ Department of Pediatric Hematology and Oncology, West China Second University Hospital, Sichuan University, Chengdu, China; ^2^ Key Laboratory of Birth Defects and Related Diseases of Women and Children (Sichuan University), Ministry of Education, Chengdu, China; ^3^ Department of Radiology, West China Second Hospital, Sichuan University, Chengdu, China; ^4^ Department of Pathology, West China Second Hospital, Sichuan University, Chengdu, China

**Keywords:** rhino-orbito-cerebral mucormycosis, very severe aplastic anemia, hematopoietic stem cell transplantation, antifungal therapy, surgical intervention

## Abstract

Rhino-orbito-cerebral mucormycosis (ROCM) is a rare and life-threatening fungal infection that predominantly affects immunocompromised individuals, such as those undergoing hematopoietic stem cell transplantation (HSCT). This report describes the case of a 10-year-old girl with very severe aplastic anemia (VSAA) who underwent an haploidentical HSCT from her father. She initially achieved successful engraftment with a donor chimerism rate of 98.25% on day 60. However, on day 65 post-transplant, she developed severe right-eye pain, progressive swelling, and visual impairment. Comprehensive evaluations, including contrast-enhanced magnetic resonance imaging (MRI) and cerebrospinal fluid (CSF) analysis, revealed extensive orbital and cerebral involvement consistent with ROCM. Intensive antifungal therapy with liposomal amphotericin B and posaconazole, along with endoscopic surgical debridement of the infected sinuses and orbital regions, was initiated. Post-treatment MRI scans demonstrated a significant reduction in cerebral edema and other abnormalities, while repeated CSF analyses confirmed the absence of fungal elements. This case underscores the critical need for early diagnosis and aggressive management of ROCM in immunocompromised patients, particularly following HSCT.

## 1 Introduction

Very severe aplastic anemia (VSAA) is a critical hematological condition characterized by a profound reduction of hematopoietic stem cells in the bone marrow, leading to severe pancytopenia (1, 2). This disorder can lead to considerable morbidity and mortality due to recurrent infections, hemorrhagic complications, and the potential development of secondary malignancies (3, 4). In the pediatric population, VSAA is often managed through hematopoietic stem cell transplantation (HSCT), which serves as a curative intervention for eligible patients (5, 6). While HSCT significantly improves survival rates and quality of life, it also carries considerable risks, particularly related to infectious complications (7–9). Immunosuppression due to conditioning regimens and graft-versus-host disease (GVHD) prophylaxis predisposes patients to a spectrum of opportunistic infections, including invasive fungal infections such as mucormycosis (10–12).

Mucormycosis, although rare, is a highly aggressive fungal infection that can have devastating consequences when it invades the central nervous system or sinuses (13, 14). This report discusses a 10-year-old girl with VSAA who underwent haploidentical HSCT from her father and subsequently developed (ROCM), a devastating complication requiring a multidisciplinary approach for diagnosis and management. This report aims to raise awareness of the clinical challenges associated with such infections and highlights the need for prompt recognition and intervention to optimize outcomes for HSCT recipients.

## 2 Case presentation

### 2.1 Medical history and clinical presentation

The patient was a 10-year-old female who initially presented with extensive skin ecchymosis accompanied by scattered petechiae. A complete blood count (CBC) revealed pancytopenia, with a white blood cell count of 3.4 × 10^9^/L, absolute neutrophil count of 0.23 × 10^9^/L, absolute reticulocyte count of 0.0113 × 10^12^/L, hemoglobin of 69 g/L, and platelet count of 73 × 10^9^/L. Bone marrow examination showed markedly decreased hematopoietic activity, with no megakaryocytes identified, leading to a diagnosis of severe aplastic anemia. Initial treatment included cyclosporine and folic acid tablets to stimulate hematopoietic recovery, along with red blood cell transfusions and platelet infusions to correct anemia and bleeding tendencies. However, the treatment response was inadequate, and the patient remained dependent on repeated transfusions. Two months following diagnosis, the patient underwent haploidentical HSCT from a related donor (patient: blood type O RhD positive; donor: blood type B RhD positive). The conditioning regimen comprised Busulfan (Bu), Fludarabine (Flud), Cyclophosphamide (CTX), Anti-thymocyte globulin (ATG), and Rituximab. Specifically, Busulfan was administered at a total dose of 0.8 mg/kg every 6 h for 2 days (days −9 and −8); Fludarabine was administered at 40 mg/m^2^/day for 5 days (days −7 to −3); Cyclophosphamide was administered at 60 mg/kg/day for 2 days (days −3 and −2); ATG was given at a total dose of 15 mg/kg over 3 days (days −4 to −2); and Rituximab was administered at 300 mg/m^2^ on day −1. During conditioning, Micafungin was administered prophylactically to prevent fungal infection.

The infused dose of CD34 + cells was 6.18 × 10⁶/kg, and mononuclear cells (MNC) dose was 8.07 × 10⁸/kg. Neutrophil and platelet engraftment occurred on days +16 and +17 post-transplant, respectively. Following transplantation, Tacrolimus and Methotrexate were administered to prevent graft-versus-host disease (GVHD), and Voriconazole was given prophylactically against fungal infections. Donor chimerism (DC) evaluations on days +21 and +60 indicated complete engraftment, with whole-blood chimerism at 98.25% and T-cell chimerism at 96.23%. However, on day +65 post-transplant, the patient began experiencing severe right ocular pain, which rapidly worsened. By day +67, the right eye exhibited significant swelling, and she reported dizziness, frontal headaches, and occasional nasal bleeding. Examination revealed elevated intraocular pressure and pronounced edema around the right eyelid. By day +72, her right eyelid displayed ptosis, and she reported blurred vision, which eventually progressed to complete vision loss in the right eye by day +74.

### 2.2 Examination and diagnosis

**Figure 1:**
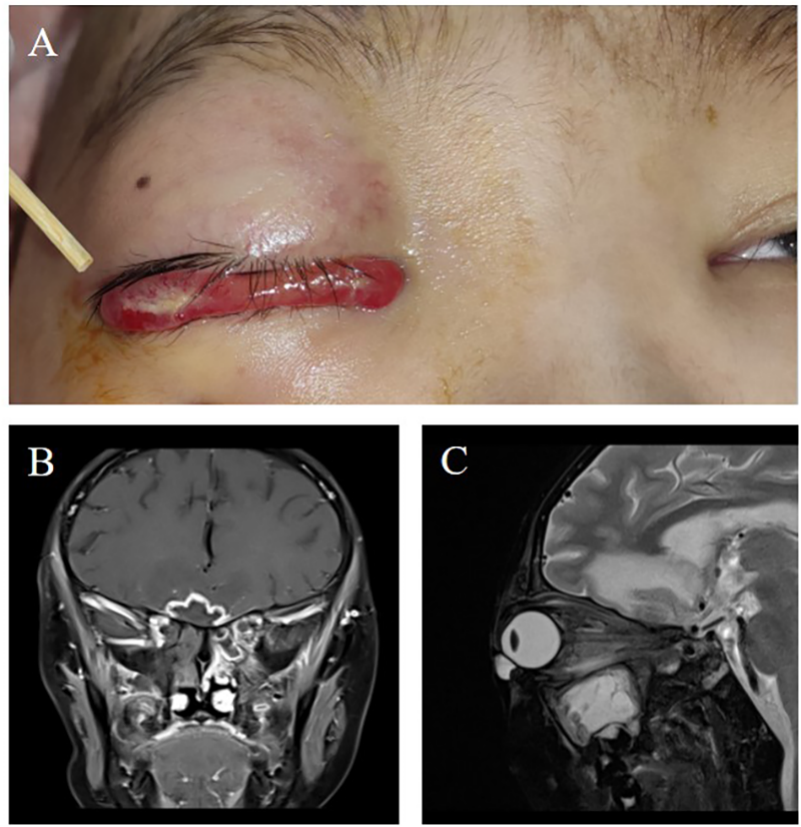
**(A)** Upon admission, the patient presented with significant redness and swelling in the right eye, accompanied by ptosis of the upper eyelid, impaired vision, and an inability to elevate the eyelid. Antifungal therapy with liposomal amphotericin B and posaconazole was initiated to address the suspected fungal infection. **(B)** Coronal view and **(C)** axial view of contrast-enhanced magnetic resonance imaging of the head showed bilateral frontal lobe parenchymal edema with abnormal signal intensities, indicative of a potential infectious process.

Upon admission for ocular symptoms, the patient was immediately initiated on broad-spectrum antibiotics, including vancomycin and meropenem, to manage potential bacterial infections. Voriconazole was concurrently administered as prophylaxis against fungal infections. Contrast-enhanced magnetic resonance imaging (MRI) of the head was performed, revealing significant swelling of the right eyelid, extraocular muscles, and surrounding soft tissues, accompanied by abnormal signals suggestive of infectious lesions (Figure 1). To further evaluate the extent of infection, metagenomic next-generation sequencing (mNGS) of cerebrospinal fluid (CSF) was conducted, confirming the presence of fungal pathogens, specifically mucormycosis, and human herpesvirus 5 (cytomegalovirus, CMV). Given the patient's positive serum CMV-DNA results, antiviral therapy with ganciclovir and foscarnet sodium was initiated, after which repeat testing confirmed clearance of CMV infection.

Given her rapid clinical deterioration and the high suspicion of invasive fungal disease, her antifungal regimen was promptly adjusted from voriconazole to liposomal amphotericin B and isavuconazole to ensure broad-spectrum coverage against potential fungal pathogens (Table 1). Multiple fundoscopic examinations revealed edema of the right optic nerve and focal hemorrhages. Soon after, the left eye began displaying similar symptoms, suggesting systemic disease involvement. Follow-up MRI showed bilateral optic nerve enlargement, abnormal signals, and ring-enhancing lesions, confirming the diagnosis of ROCM.

**Table 1:** Antifungal agents, doses, and duration.

Antifungal agent	Dose	Duration
Voriconazole	8 mg/kg every 12 h	Oct 18–Oct 22, 2023
Liposomal Amphotericin B	10 mg/kg/day	Oct 23–Dec 15, 2023
Isavuconazole	10 mg/kg/day	Oct 23–Dec 15, 2023
Amphotericin B	1.5 mg/kg/day	Dec 16, 2023–Jan 5, 2024
Posaconazole	6 mg/kg/day	Dec 16, 2023–Dec 16, 2024

### 2.3 Treatment and management

Managing ROCM in this immunocompromised patient required a collaborative, multidisciplinary approach due to the aggressive nature of the infection. After extensive discussions among specialists, a revised treatment strategy was developed, which included continuing liposomal amphotericin B and isavuconazole. Localized therapy was also implemented, including the administration of amphotericin B liposomal spray directly to the infected areas. In addition to pharmacological interventions, endoscopic surgical debridement was essential for removing necrotic tissue in the nasal and orbital cavities.

**Figure 2:**
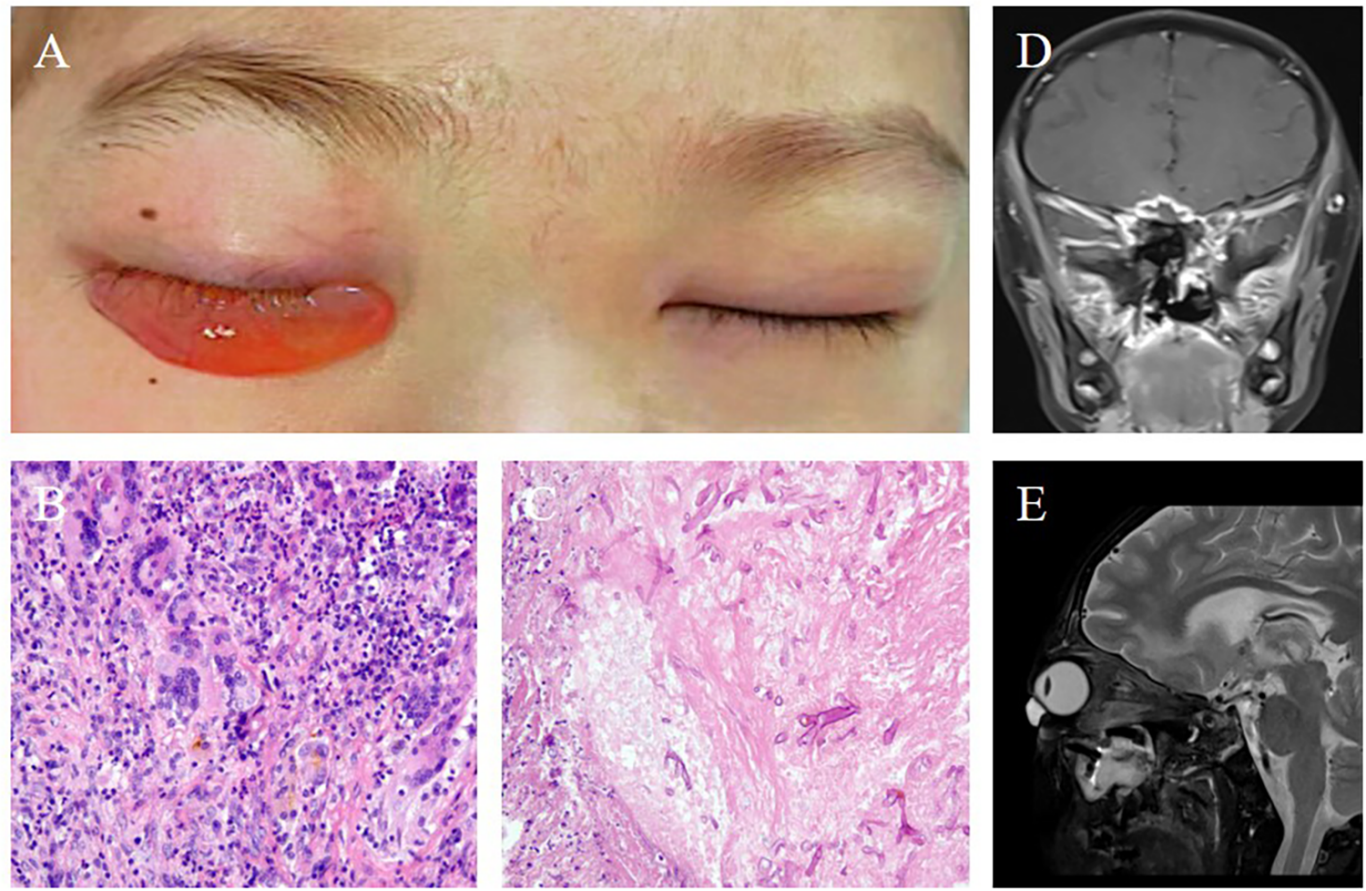
**(A)** Following nasal endoscopic surgery by the otolaryngology team, persistent marked edema was observed in the right conjunctiva, while the swelling in the left eye had improved. **(B)** Histopathological examination of the excised tissue revealed a multinucleated giant cell response, suggestive of a fungal infection (200× magnification). **(C)** Further histopathological analysis identified chronic inflammatory changes with fungal hyphae within necrotic tissue (200× magnification). **(D)** Coronal and **(E)** axial views of contrast-enhanced magnetic resonance imaging of the head, performed 9 weeks post-surgery, displayed radiologic findings consistent with the nasal endoscopic procedure, confirming the ongoing resolution of the infection.

During the procedure, a large fungal mass was identified in the right nasal cavity, alongside extensive mucosal necrosis. The left nasal cavity showed purulent secretions, requiring careful debridement. Histopathological analysis of the debrided tissue confirmed fungal sinusitis with fungal hyphae consistent with mucormycosis (Figure 2). After surgery, the patient continued with antifungal therapy, including liposomal amphotericin B and isavuconazole, along with sinus irrigation using bicarbonate-diluted solutions to facilitate drainage and infection control. Further, the girl also underwent eyelid cleft operation, conjunctival mass excision and conjunctival cystoplasty due to eyelid edema.

### 2.4 Follow-up

**Figure 3:**
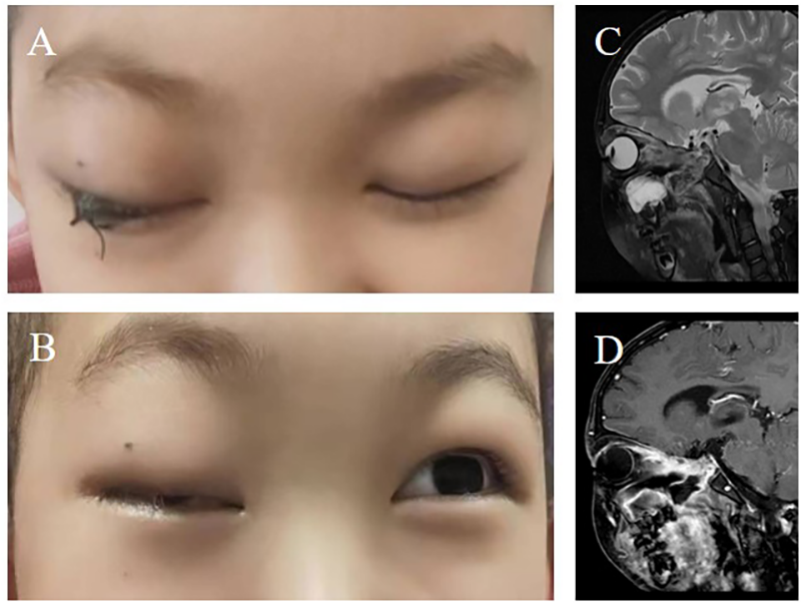
**(A)** Photographic representation of the tarsorrhaphy surgery performed to address the patient's visual complications. **(B)** At the 24-week postoperative follow-up, both eyes exhibited a lack of light perception, indicating persistent visual impairment. **(C)** Coronal and **(D)** axial views of contrast-enhanced magnetic resonance imaging of the head, conducted 24 weeks after surgery, provided a radiologic assessment of the patient's condition, showing the extent of recovery and ongoing changes in the affected areas.

Following surgical intervention and subsequent treatment modifications, the patient was closely monitored for clinical progress. MRI scans of the head performed at 9 and 24 weeks post-operatively revealed marked reductions in bilateral frontal lobe swelling and abnormal signal intensity, demonstrating a favorable response to intensive antifungal therapy. Serial lumbar punctures were conducted to evaluate residual fungal elements in the cerebrospinal fluid (CSF), with all test results consistently negative, indicating effective infection control. The patient's ocular symptoms gradually improved, and partial vision recovery was documented during follow-up visits (Figure 3). Due to financial constraints, after nearly 2 months of treatment with liposomal amphotericin B combined with isavuconazole, the regimen was changed to amphotericin B combined with posaconazole for 1 month, followed by oral posaconazole maintenance therapy, which continued until 1-year post-treatment (Table 1).

## 3 Discussions

ROCM is a highly aggressive, often fatal fungal infection primarily caused by species within the *Mucorales* family, particularly *Rhizopus* and *Mucor* (15–17). This infection typically affects immunocompromised patients, such as those undergoing HSCT, due to their reduced immune defense capabilities (5, 11, 12). We presented a complex case of ROCM in a 10-year-old girl following haploidentical HSCT for VSAA. This case underscores the complexity of diagnosing ROCM and the challenge of formulating effective treatment strategies, especially in high-risk pediatric patients undergoing mismatched transplants.

*Mucorales* fungi have a distinct affinity for vascular tissues, leading to angioinvasion, thrombosis, and subsequent tissue necrosis (18). In immunocompromised patients, such as those post-HSCT, these organisms thrive due to impaired phagocytic cell function, delayed immune reconstitution, and the immunosuppressive effects of medications used to prevent GVHD (11, 19). In the presented case, the patient received a 7/12 HLA-matched HSCT, which resulted in delayed engraftment and extended immunosuppression. Additionally, prophylactic use of tacrolimus and methotrexate against GVHD further intensified her immunosuppressive state, significantly increasing susceptibility to opportunistic infections like mucormycosis. This case emphasizes the cumulative impact of these risk factors in predisposing post-HSCT patients to invasive fungal infections (IFIs), specifically ROCM.

Early diagnosis of ROCM is challenging due to its nonspecific initial symptoms, which can often be mistaken for bacterial or viral infections (15, 20). In this case, the initial symptoms of orbital pain, redness, and swelling might have been interpreted as typical post-transplant infections. However, the rapid symptom progression, including visual impairment, underscored the need for heightened clinical suspicion of ROCM in high-risk patients. Imaging techniques, such as MRI, are critical for identifying fungal invasion in the orbital and cranial regions, although findings are often nonspecific and require confirmation through microbiological or histopathological evidence (21). Here, MRI revealed optic nerve thickening, retrobulbar tissue involvement, and abnormal signals in the frontal lobes, suggesting invasive fungal spread. The mNGS of the CSF provided crucial pathogen identification, confirming the presence of *Mucorales* and human herpesvirus 5, which guided targeted antifungal therapy. Thus, mNGS is increasingly recognized as a valuable diagnostic tool for rapid and accurate pathogen identification in IFIs, especially in cases where fungal cultures may be unreliable or delayed.

The management of ROCM requires an aggressive, multimodal approach, including antifungal therapy, surgical debridement, and supportive care (14, 15, 17, 22). In this case, initial antifungal therapy with voriconazole proved ineffective against Mucorales. Following pathogen identification, the antifungal regimen was promptly modified to include liposomal amphotericin B combined with isavuconazole. Due to financial constraints, after approximately 2 months of treatment with liposomal amphotericin B and isavuconazole, therapy was transitioned to amphotericin B combined with posaconazole for an additional month. After a total of 3 months of intravenous antifungal therapy, treatment was changed to oral posaconazole, maintained for up to 1 year. The extended antifungal regimen ensured thorough eradication of the fungal infection and significantly improved long-term clinical outcomes. Concurrent use of multiple antifungal agents, such as amphotericin B combined with posaconazole or isavuconazole, is recommended in severe cases to enhance efficacy and minimize the risk of resistance (13, 23). Surgical intervention plays a crucial role in ROCM management, as antifungal agents exhibit limited penetration into necrotic tissues (22, 24). In this patient, aggressive surgical debridement was performed, including the removal of necrotic tissues from the nasal cavity, paranasal sinuses, and orbit, along with optic nerve decompression, significantly reducing the fungal load and restricting further spread. However, the timing and extent of surgery must be carefully balanced against the risk of surgical morbidity, given the rapid progression of ROCM, which can lead to significant intracranial complications.

Given the high mortality associated with ROCM, preemptive or prophylactic antifungal therapy could benefit patients undergoing HSCT, particularly those receiving mismatched HLA transplants. This case suggests a potential need for such preventive measures during the critical early post-transplant period to reduce fungal infection risk in pediatric HSCT recipients. Furthermore, multidisciplinary collaboration is essential in managing complex ROCM cases. In this instance, coordinated care among hematology, infectious disease, ophthalmology, radiology, and surgical teams ensured timely and appropriate interventions. Regular monitoring through imaging and microbiological testing is also vital for early detection of disease progression, particularly in patients with delayed immune reconstitution.

Overall, this case emphasizes the importance of prompt diagnosis and aggressive management of mucormycosis, particularly in immunocompromised patients following HSCT. The combination of timely antifungal therapy, surgical intervention, and collaborative multidisciplinary care significantly contributed to the favorable outcome in this patient's case, despite the challenging and potentially fatal nature of the infection.

## 4 Conclusions

This case highlights the significant challenges associated with managing ROCM in pediatric HSCT recipients, particularly in the context of ABO-mismatched transplantation. The rapid progression of ROCM, coupled with limited therapeutic options for central nervous system involvement, underscores the urgent need for ongoing research into improved prophylactic and therapeutic strategies. Enhanced fungal prophylaxis, carefully tailored immunosuppressive protocols, and a multidisciplinary approach to patient care are essential to mitigate the devastating impact of ROCM in immunocompromised children.

## Data Availability

The original contributions presented in the study are included in the article/Supplementary Material, further inquiries can be directed to the corresponding author.
